# The Extracellular Matrix of *Candida albicans* Biofilms Impairs Formation of Neutrophil Extracellular Traps

**DOI:** 10.1371/journal.ppat.1005884

**Published:** 2016-09-13

**Authors:** Chad J. Johnson, Jonathan Cabezas-Olcoz, John F. Kernien, Steven X. Wang, David J. Beebe, Anna Huttenlocher, Hamayail Ansari, Jeniel E. Nett

**Affiliations:** 1 Department of Medicine, University of Wisconsin, Madison, Wisconsin, United States of America; 2 Department of Biomedical Engineering, University of Wisconsin, Madison, Wisconsin, United States of America; 3 Department of Medical Microbiology and Immunology, University of Wisconsin, Madison, Wisconsin, United States of America; 4 Department of Pediatrics, University of Wisconsin, Madison, Wisconsin, United States of America; University of Birmingham, UNITED KINGDOM

## Abstract

Neutrophils release extracellular traps (NETs) in response to planktonic *C*. *albicans*. These complexes composed of DNA, histones, and proteins inhibit *Candida* growth and dissemination. Considering the resilience of *Candida* biofilms to host defenses, we examined the neutrophil response to *C*. *albicans* during biofilm growth. In contrast to planktonic *C*. *albicans*, biofilms triggered negligible release of NETs. Time lapse imaging confirmed the impairment in NET release and revealed neutrophils adhering to hyphae and migrating on the biofilm. NET inhibition depended on an intact extracellular biofilm matrix as physical or genetic disruption of this component resulted in NET release. Biofilm inhibition of NETosis could not be overcome by protein kinase C activation via phorbol myristate acetate (PMA) and was associated with suppression of neutrophil reactive oxygen species (ROS) production. The degree of impaired NET release correlated with resistance to neutrophil attack. The clinical relevance of the role for extracellular matrix in diminishing NET production was corroborated in vivo using a rat catheter model. The *C*. *albicans pmr1Δ/Δ*, defective in production of matrix mannan, appeared to elicit a greater abundance of NETs by scanning electron microscopy imaging, which correlated with a decreased fungal burden. Together, these findings show that *C*. *albicans* biofilms impair neutrophil response through an inhibitory pathway induced by the extracellular matrix.

## Author Summary

*Candida spp*. avidly adhere to medical device surfaces, forming resilient, drug-tolerant biofilms which are encased in a protective extracellular matrix. These infections are notoriously difficult to eradicate and little is known about how they evade host defenses. Here we show a mechanism by which *C*. *albicans* biofilms inhibit the activity of neutrophils, leukocytes critical for protection from *Candida*. When exposed to biofilm, neutrophils fail to release neutrophil extracellular traps (NETs), web-like structures of DNA, histones, and proteins capable of fungal killing. The biofilm extracellular matrix appears to have a major role in this inhibition, as disruption of matrix polysaccharides by physical or genetic means reverses the impairment. *C*. *albicans* biofilms block neutrophil production of reactive oxygen species (ROS), a signaling pathway involved in NET production. Impaired NET release results in decreased fungal killing in vitro and in an animal model of biofilm infection. This represents a novel mechanism of immune evasion specific to the biofilm mode of growth.

## Introduction

*Candida albicans* is a widespread nosocomial fungal pathogen and frequent cause of bloodstream infection [1]. One of the most common risk factors of invasive candidiasis is medical device placement, with nearly 80% of patients having vascular catheters [2]. On these and other medical devices, *C*. *albicans* adopts a biofilm lifestyle. As an adherent microbial community, *Candida* is capable of withstanding conventional antifungals and host defenses [3–9]. This biofilm mode of growth presents a significant obstacle for effective treatment of candidiasis. Despite advancements in antifungal therapies and diagnostics, the mortality associated with invasive candidiasis remains exceedingly high, near 30%, and is even higher for patients with biofilm-infected devices that are retained [1, 2]. Little is known about the host immune system response to device-associated *Candida* infection and why these biofilms are so resilient.

One defining biofilm characteristic is the production of a protective extracellular matrix [10]. Recent analysis of *C*. *albicans* matrix identified α-mannan and β-1,6-glucan as the most abundant polysaccharides [11]. However, when compared to cell wall polysaccharides, striking differences were noted in their structures. For example, the α-1,2 branched α -1,6-mannan found in the matrix contained over 50-fold more residues than cell wall mannan [12]. Also, the mannan co-isolated with linear β-1,6-glucan, a structure distinct from the branched structure described for cell wall glucan [13]. A complex of these unique matrix polysaccharides was shown to encase the cells, promoting drug resistance and providing biofilm structure [14]. Leukocyte recognition of biofilms likely involves these and other matrix components, which are shielding the cell wall.

Neutrophils are an essential host component required for control of numerous invasive fungal infections, including invasive candidiasis [15–18]. Patients with neutropenia are particularly prone to severe, life-threatening disease and those who remain neutropenic are at risk for relapse [19]. A recent study examining the host response to device-associated *Candida* biofilms identified neutrophils as the primary biofilm-associated leukocyte [20]. This finding was observed for multiple animal models mimicking clinically relevant biofilm infections, including a vascular catheter, a urinary catheter, and a denture-associated *C*. *albicans* infection [20]. However, it remains a mystery how neutrophils respond to these common biofilm infections and why this response is ineffective.

While neutrophils are capable of phagocytosing *Candida* yeast, the larger hyphal forms trigger the release of neutrophil extracellular traps (NETs) [21, 22]. These protruding fibrillary structures are composed of granular proteins and histones on a web of DNA [23]. NETs kill both yeast and hyphal forms of *C*. *albicans* in vitro, with antifungal activity linked to NET-bound calprotectin, which chelates cations required for proliferation of microorganisms [21, 24]. NETs are critical for control of in vivo infection due to their activity against hyphae, which are too large to be ingested [22]. It would reason that NETs would be an ideal response against aggregated biofilm communities. In the current investigation, we show that *C*. *albicans* biofilms impair NET release as a mechanism to resist killing by neutrophils. We describe how the biofilm architecture and matrix drive the inhibition of this important immune function.

## Results

### *C*. *albicans* biofilms impair release of NETs and resist killing by neutrophils

**Fig 1:**
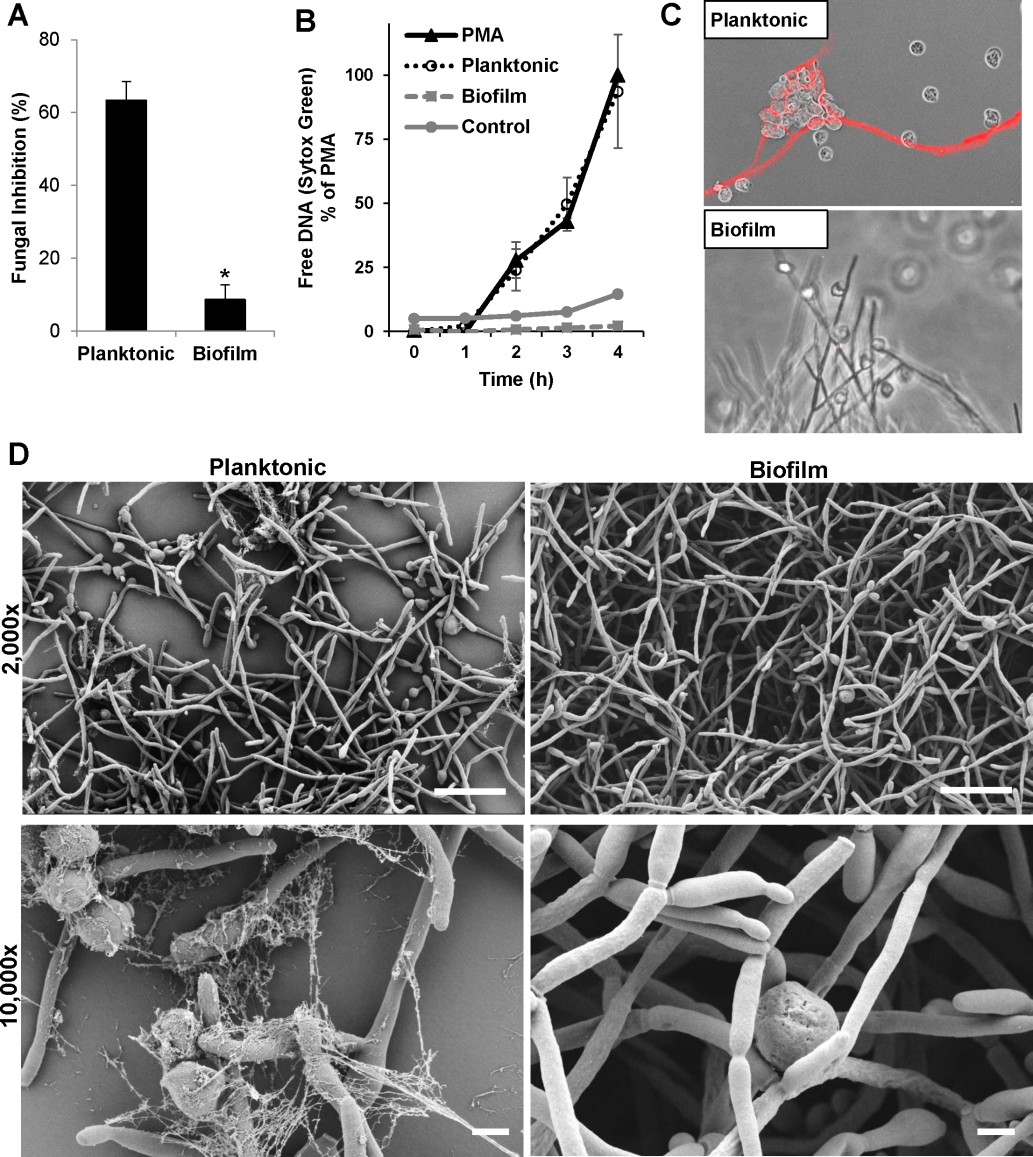
*C*. *albicans* biofilms resist killing by neutrophils and impair release of NETs. (A) Planktonic and biofilm *C*. *albicans* were co-cultured with human neutrophils at an effector:target ratio of 1:1 and fungal inhibition was estimated by an XTT assay after neutrophil lysis. Neutrophils have very little activity against biofilms, but strongly inhibit planktonic *Candida*, *n = 11*. (B) The rate of NET release was estimated by Sytox Green detection of free DNA. Planktonic *Candida* generated high fluorescence, representing NET release, comparable to the levels induced by PMA. In contrast, biofilms did not produce fluorescence, similar to the neutrophil only control, *n = 3*. (C) Following co-culture with neutrophils for 4 h, the neutrophil response to planktonic and biofilm *C*. *albicans* was visualized by immunofluorescence using an anti-citrullinated H4 antibody. NETs were observed in response to planktonic *Candida*, but were rarely produced in response to biofilm. (D) By scanning electron microscopy, thread-like NETs covered planktonic cells after a 4 h co-culture with neutrophils. In contrast, neutrophils exposed to biofilm appeared rounded, with few NETs released. Measurement bars represent 20 μm and 2 μm for 2,000x and 10,000x images, respectively. *P<0.*05* Error bars represent SEM.

We examined neutrophil-biofilm interactions by co-culturing isolated human neutrophils with *C*. *albicans* biofilms. We found biofilms to be over 5-fold more resistant to killing by neutrophils when compared to planktonic cells (Fig 1A). This is consistent with prior studies showing that biofilms resist neutrophil killing [7, 25, 26]. To examine if this impairment in neutrophil function may be due to a difference in release of NETs, we performed several complementary experiments [21, 23, 27, 28]. First, we utilized the cell-impermeable Sytox Green dye to measure free DNA as an estimate of NETs [27]. Over the course of 4 hours, planktonic *C*. *albicans* triggered elevated free DNA, increasing more than 20-fold, consistent with release of NETs (Fig 1B). This rise mirrored the response to phorbol myristate acetate (PMA), a potent inducer of NET release. In comparison, no elevation of free DNA was observed in response to biofilms. To determine if the free DNA we were detecting was due to NET release, we utilized immunofluorescence imaging with labeling of citrullinated histones, a modification present in NETs (Fig 1C) [29]. Upon co-culture of planktonic *Candida* and neutrophils, thin strands of immunofluorescence were observed to encompass groups of *Candida*, consistent with the presence of NETs. However, these were rarely visualized when neutrophils were exposed to *C*. *albicans* biofilms. We further examined the neutrophil response by scanning electron microscopy (Fig 1D) [27]. After a 4 h exposure to planktonic cells, numerous web-like fibrillary structures coated the fungi, consistent with the release of NETs. The structures were spread over yeast cells as well as the hyphae, which had developed in response to the temperature and media of the assay. In contrast, upon neutrophil exposure to biofilm, neutrophils associating with the biofilm appeared rounded.

**Fig 2:**
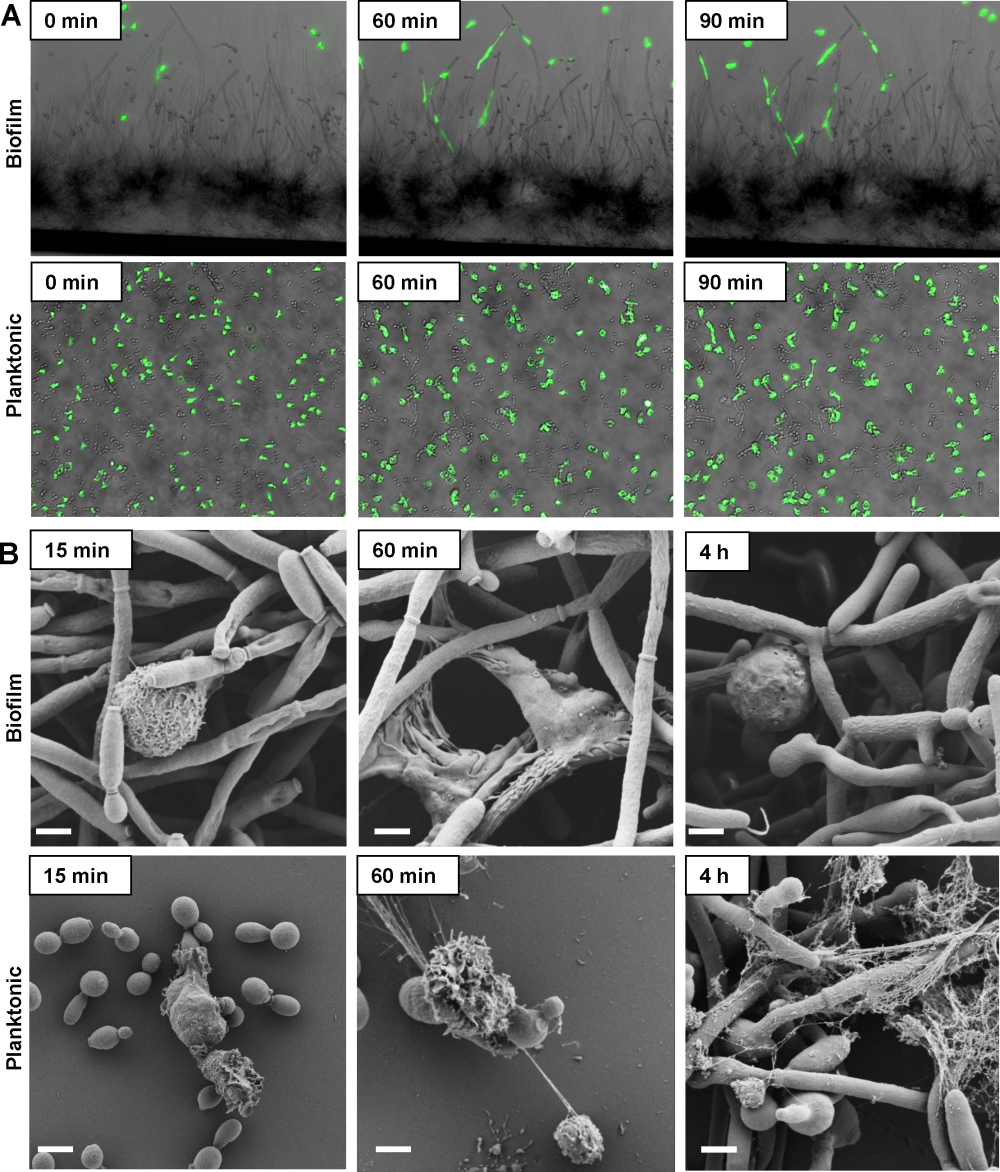
Neutrophils adhere to *C*. *albicans* biofilm. (A) Fluorescently-labeled neutrophils were added to microfluidic channels with *C*. *albicans* biofilms, which had been propagated on the sidewall, or to planktonic *C*. *albicans*. Over the course of 90 min, neutrophils migrated to the biofilm, adhered, and extended over the surface of the hyphae. In contrast, neutrophils engulfed planktonic *C*. *albicans* and did not appear to elongate. (B) Neutrophil interactions with *C*. *albicans* were examined by scanning electron microscopy. In response to biofilms, neutrophils initially adhered to hyphae, then elongated with extended filopodia, and ultimately appeared rounded. Upon co-culture with planktonic cells, NETs developed over this 4 h time period. Measurement bars represent 2 μm for 10,000x images. Representative data are shown for experiments performed with neutrophils from at least 3 different donors on different days.

The rounded neutrophil appearance accompanied by the lack of NETs in response to biofilm prompted us to examine if biofilms were recognized by neutrophils. To investigate this possibility, we observed the neutrophil response to *C*. *albicans* biofilms growing in microfluidic devices with time lapse microscopy. *C*. *albicans* biofilms were propagated on the channel sidewall and neutrophil migration was imaged over a course of 90 min (Fig 2A and S1 File). Neutrophils were observed to migrate and adhere to the biofilm with a predilection for hyphae. Upon contact, the neutrophils elongated and migrated along hyphae. However, they appeared to stall before reaching the deepest basal biofilm layer. In contrast to this, neutrophils engulfed planktonic *Candida* and did not appear to elongate (Fig 2A and S2 File). We further explored this interaction with scanning electron microscopy (Fig 2B). Neutrophils were observed to release NETs in response to planktonic cells over the course of 4 h. Upon biofilm presentation, neutrophils were rounded and beginning to adhere to hyphae. By 1 h, neutrophils had clearly fastened to hyphae and exhibited extended filopodia, often stretching over multiple hyphae. However, by 4 h, the neutrophils again appeared rounded and inactive. Time lapse imaging confirmed that the neutrophils remained viable at this time point, excluding the propidium iodide stain (S3 File). Taken together, these time course studies show an active interaction between *C*. *albicans* biofilms and neutrophils, where neutrophils adhere to the biofilm, elongate, and migrate. However, this process ends without NET release.

### NET inhibition by biofilm is contact-dependent and requires intact architecture

**Fig 3:**
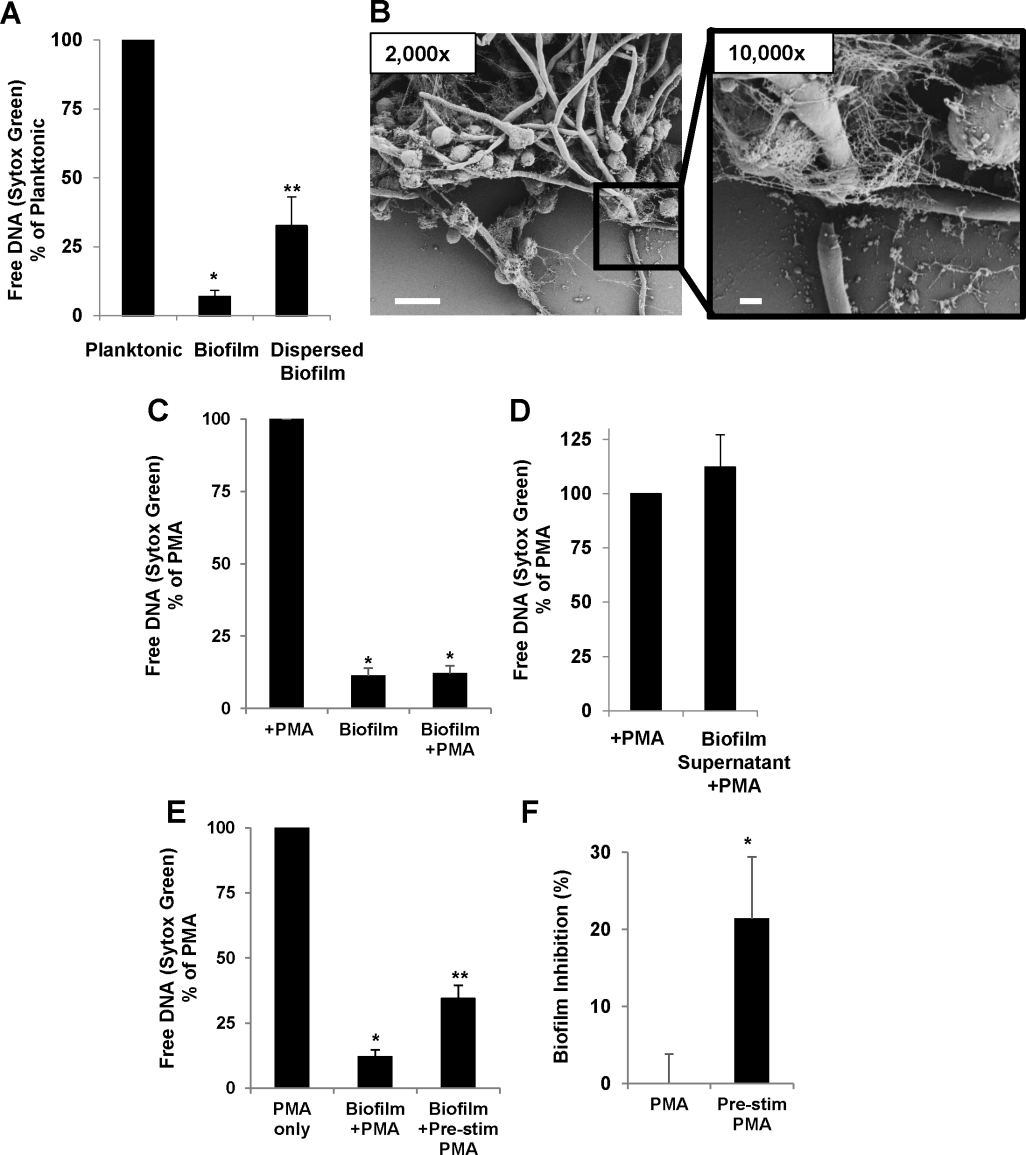
NET inhibition by *C*. *albicans* is dependent on intact biofilm architecture and is not due to a soluble factor. (A) Planktonic, biofilm, and partially dispersed biofilms were co-cultured with human neutrophils for 4 h and NET release was estimated by Sytox Green detection of free DNA. Dispersed biofilms induced NETs, marked by elevated Sytox Green, *n = 6*. (B) By scanning electron microscopy, NETs were triggered in response to disrupted biofilm, indicating intact structure is necessary for NET inhibition. The measurement bars represent 20 μm and 2 μm for the 2,000x and 10,000x images, respectively. (C) *C*. *albicans* biofilms and PMA (an inducer of NETs), alone and in combination were incubated with neutrophils for 4 h and NET release was estimated by Sytox Green. While PMA alone generated a robust NET response, the combination of PMA and biofilm did not trigger DNA release. (D) Supernatants from biofilms (24 h) were collected at 2 h and added to Sytox Green assays in combination with PMA. Biofilm supernatants alone did not block PMA induction of NETs, *n = 5*. (E) Neutrophils were pre-stimulated (pre-stim) with PMA to induce NETs for 90 min prior to addition to biofilms. While pre-stimulation increased NET release, biofilms still inhibited NETs compared to the PMA only control, *n = 4*. (F) The activity of pre-stimulated neutrophils against biofilms was estimated by XTT assay following lysis of neutrophils. Neutrophils pre-stimulated to induce NETs inhibited biofilms *n = 4*. *P<0.*05* compared to reference, **P<0.*05* compared to *. Error bars represent SEM.

Biofilms are heterogeneous communities of adherent cells encased in an extracellular matrix. We questioned if this covering may be a critical factor for the altered recognition and impairment of NET release. To test this, we physically dispersed *C*. *albicans* biofilms and examined the neutrophil response. In contrast to intact biofilm, which did not elicit NET release, the dispersed biofilms triggered a 5-fold increase in free DNA release by Sytox Green (Fig 3A). We also used scanning electron microscopy to examine NET release in response to disrupted biofilm. Consistent with the Sytox Green assay results, many NETs were observed to associate with the edge of the biofilm that had been disrupted (Fig 3B). This was in stark contrast to the intact biofilm, where neutrophils appeared rounded and very few neutrophils had released NETs (Fig 1D).

The observation that dispersed or disrupted biofilm induced NET release suggested that inhibition of NET release may be dependent on neutrophil contact with the biofilm. To further test this hypothesis and exclude the possibility of a soluble inhibitor of NETs, we investigated the impact of biofilm supernatants on neutrophil function. We utilized PMA, an inducer of NET release, and examined NET production in the presence of biofilm supernatants [30]. While exposure to PMA alone generated NETs, *C*. *albicans* biofilms inhibited PMA-induced NETs (Fig 3C). This inhibition was not reproduced when biofilm supernatants were combined with PMA, suggesting that the phenotype is not due to a soluble factor (Fig 3D). Instead, the finding is consistent with a contact-dependent mechanism of NET suppression.

### Pre-stimulated NETs exhibit anti-biofilm activity

Our studies show impairment of NET release by *C*. *albicans* biofilms. We next sought to determine if NETs were capable of killing biofilm-associated cells. To examine if NETs would demonstrate anti-biofilm activity, we utilized PMA for NET induction. When neutrophils were simultaneously presented with both PMA and biofilm, NETs were inhibited (Fig 3C). However, neutrophils could be induced to produce NETs by pre-stimulation with PMA prior to addition to the biofilm for 90 min (Fig 3E). Even with this pre-stimulation step, biofilms still demonstrated significant NET inhibition, with NETs only reaching approximately one third of the levels of PMA alone. When applied to biofilms, these pre-stimulated neutrophils exhibited anti-biofilm activity. Collectively, these studies show *C*. *albicans* biofilms inhibit NET release, but if this can be overcome, NETs would likely be an effective killing mechanism against biofilms.

### *C*. *albicans* biofilm determinant of NET inhibition

To uncover factors governing *C*. *albicans* biofilm inhibition of NET release, we utilized a library of strains with genetic disruption of β-1,6 glucan and mannan synthesis and processing pathways (S1 Table) [14]. This library had been previously constructed to identify genes involved in the synthesis of the *C*. *albicans* extracellular matrix, with candidate genes selected based on the chemical structure of the abundant mannan-glucan complex [11]. We screened a total of 36 *C*. *albicans* mutants, including 30 with genetic disruption in mannan pathways and 6 with disruption in β-1,6 glucan pathways. *C*. *albicans* strains were grown as biofilms and Sytox Green was used to estimate NET release. The screen identified 14 strains which elicited fluorescence at least 1.5 fold higher than the reference strain, demonstrating that these pathways likely play a role in the suppression of NETs by *C*. *albicans* biofilms (S2 Table). Considering 10 of these strains had disruption in mannan pathways, we chose this mutant subset for further investigation.

### Biofilm inhibition of NETs is dependent on *C*. *albicans* mannosylation

**Fig 4:**
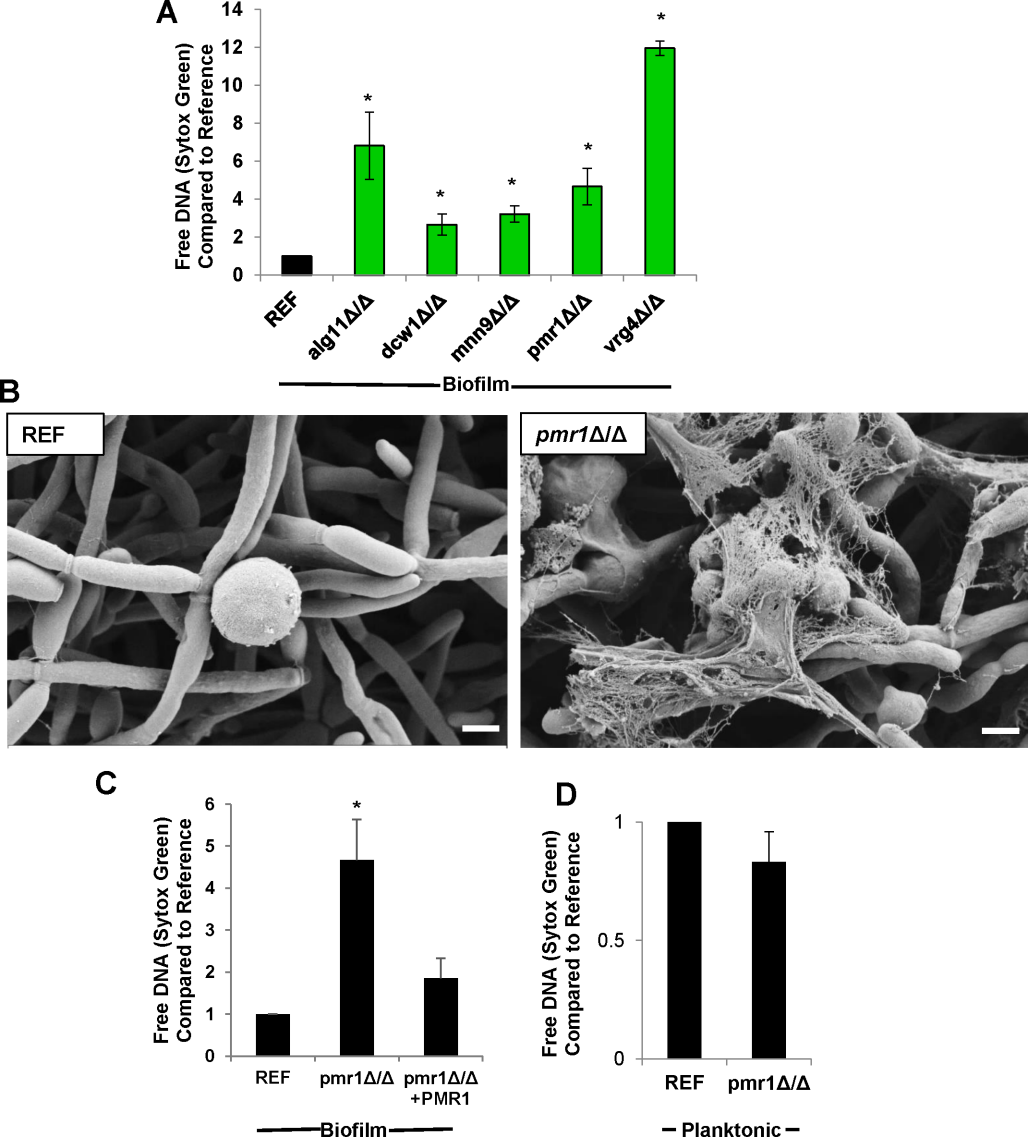
Biofilm inhibition of NETs is dependent on *C*. *albicans* mannosylation. (A) NET release was estimated by Sytox Green after neutrophils were exposed to *C*. *albicans* biofilms for 4 h. Five mutants with disruption of mannan pathways triggered NET release in response to neutrophils from multiple donors, *n = 4*. (B) By scanning electron microscopy, neutrophils exposed to the reference strain biofilm appeared rounded, while the *pmr1Δ/Δ* biofilm elicited NETs. Measurement bars represent 2 μm for 10,000x images. (C) Complementation of the *pmr1Δ/Δ* mutant mostly restored the NET inhibition phenotype *n = 4*. (D) NET release was measured in response to planktonic *C*. *albicans*. In contrast to the response to biofilms, no significant difference was observed between *pmr1Δ/Δ* and the reference strain, *n = 6*. *P<0.*05* compared to reference. Error bars represent SEM.

For the *C*. *albicans* mannan mutants selected from our screen, we confirmed the phenotype of the triggering of NETs for at least 3 neutrophil donors. Five of the mutant strains consistently elicited higher NET release as estimated by Sytox Green (Fig 4A). The four strains with the most pronounced phenotype (*alg11*Δ/Δ, *mnn9*Δ/Δ, *pmr1*Δ/Δ and *vrg4*Δ/Δ) had previously been shown to be involved in production of the extracellular matrix mannan-glucan complex, suggesting a role for this polysaccharide in impairment of NET release [14]. However, a biofilm defect was noted for two of these strains (*alg11*Δ/Δ and *vrg4*Δ/Δ) in our model (S1 Fig). Therefore, we chose to focus on *pmr1*Δ/Δ, the mutant strain which elicited the highest NET release, while still forming a mature biofilm. *PMR1* encodes a P-type Ca^2+^/Mn^2+^-ATPase which transports ions into the Golgi [31]. As mannosyltransferases require Mn^2+^, disruption of *PMR1* impacts both O- and N-mannan, resulting in truncated cell wall mannans. During biofilm growth, *PMR1* is also required for production of extracellular matrix polysaccharides, including a large mannan structure which ultimately associates with glucan [14]. Consistent with the role of *PMR1* in biofilm matrix production, we found the *PMR1* transcript level to be more abundant during biofilm growth when compared to planktonic conditions (2.7 +/- 0.3-fold by RT-PCR).

By scanning electron microscopy, the *pmr1*Δ/Δ biofilm had an appearance very similar to the reference strain consisting primarily of hyphae with few yeast forms (Fig 4B and S1 Fig). Consistent with results of the Sytox Green assays, an increase in NETs was observed in response to the *pmr1*Δ/Δ biofilm, when compared to the reference strain biofilm (Fig 4B). The mutant phenotype was mostly reversed with complementation of a single allele (Fig 4C). The *pmr1*Δ/Δ*+PMR1* biofilm triggered fewer NETs than the *pmr1*Δ/Δ biofilm, approaching the number for the reference strain. This is consistent with prior studies examining *PMR1* that have demonstrated a similar pattern, with incomplete phenotype restoration upon integration of a single allele [14, 32]. To assess if the *pmr1*Δ/Δ phenotype was unique to the biofilm mode of growth, we examined the neutrophil interactions with planktonic cells. Similar to the reference strain, the *pmr1*Δ/Δ mutant elicited NET release on co-culture (Fig 4D). This suggests that the increased NET induction by the *pmr1*Δ/Δ biofilm is not due to a cell wall defect and it is specific to the biofilm mode of growth.

### Mechanism of *C*. *albicans* biofilm impairment of NETs

**Fig 5:**
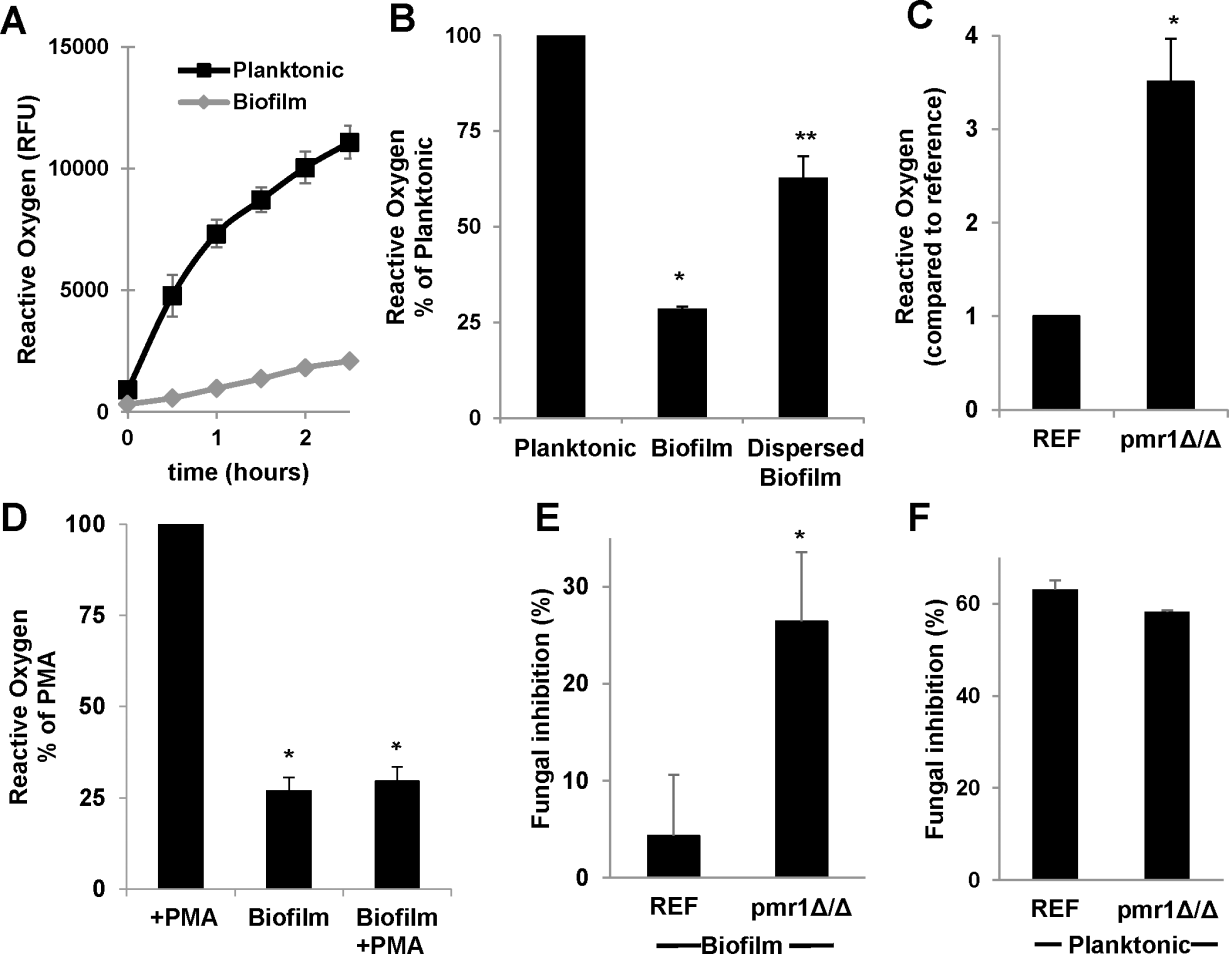
C. *albicans* biofilm extracellular matrix inhibits neutrophil production of ROS and protects against neutrophil activity. (A-D) Neutrophils were pre-labeled with CM-H2DCFDA and exposed to *C*. *albicans* biofilms in the presence or absence of PMA and ROS was measured by fluorescence. (A) Neutrophils produced higher levels of ROS in response to planktonic cells over the course of 3 h, *n = 4*, *representative data with SD shown*. (B) Dispersion of biofilms increased neutrophil production of ROS *n = 5*, *SEM shown*. (C) Neutrophils generated increased ROS in response to the *C*. *albicans pmr1Δ/Δ* mutant biofilm, several fold above the reference strain, *n = 9*, *SEM shown*. (D) *C*. *albicans* biofilms inhibited ROS production in response to PMA stimulation *n = 11*, *SEM shown*. (E-F) *C*. *albicans* biofilms and planktonic cells were co-cultured with human neutrophils and fungal killing was estimated by an XTT assay after neutrophil lysis. Neutrophils exhibited increased activity against the *pmr1Δ/Δ* mutant biofilm compared to the reference strain (E) but no difference was observed for planktonic cells (F), *n = 6 and 4*, *SEM shown*. *P<0.*05* compared to reference.

The generation of ROS and its subsequent processing by myeloperoxidase are key steps in production of NETs [33, 34]. ROS triggers the release of neutrophil elastase from granules to the cytosol where it degrades F-actin to halt migration before translocating to the nuclease for partial cleavage of histones, allowing for chromatin decondensation. To test the possibility that biofilm formation may be disrupting this process, we measured neutrophil production of ROS in response to *C*. *albicans* biofilms. Neutrophils were pre-treated with a free radical sensor (CM-H2DCFDA) and ROS was measured by fluorescence. Upon exposure to biofilms alone, neutrophils generated approximately 4-fold less ROS compared to a similar number of planktonic cells (Fig 5A and 5B). Disruption of the biofilm architecture through biofilm dispersion increased ROS production to more than 60% of that measured in response to planktonic *C*. *albicans*. We additionally examined ROS in response to matrix disruption using the *pmr1*Δ/Δ mutant. In contrast to the reference strain, the *pmr1*Δ/Δ biofilm allowed for ROS activation, inducing over 3-fold higher ROS levels (Fig 5C).

We further examined the mechanism of neutrophil impairment using PMA to induce NETs. PMA is an activator of protein kinase C, which mediates activation of NADPH, an enzyme complex responsible for ROS generation during NETosis [35]. In agreement with prior investigations, PMA alone induced neutrophil generation of ROS (Fig 5A) [34, 36]. However, *C*. *albicans* biofilm abrogated PMA-inducted ROS. This finding is consistent with the induction of an inhibitory pathway of NADPH oxidase which is downstream of protein kinase C activation (Fig 5D). Collectively, these studies show *C*. *albicans* biofilms likely impair NET release by inhibiting NADPH-oxidase and ROS production in a manner dependent on intact biofilm structure and matrix production.

### Impaired NET release protects biofilms from neutrophil killing

To examine if the increased NET release associated with the *pmr1*Δ/Δ mutant resulted in increased anti-*Candida* activity, we examined the *Candida* burden following co-culture with neutrophils. The viable fungal burden was estimated by XTT assay after lysis of neutrophils. While neutrophils demonstrated minimal activity against the reference strain biofilm, the *pmr1*Δ/Δ biofilm burden was reduced by approximately 25% (Fig 5E). This degree of killing is similar to the results from the studies adding pre-stimulated neutrophils to biofilm (Fig 3F). Neutrophils were similarly effective against both the reference strain and *pmr1*Δ/Δ planktonic cells (Fig 5F). Taken together, these studies show *PMR1* is required for impairment of NET release and the resistance to killing by neutrophils during biofilm growth.

### Biofilm mannosylation dampens NET release and enhances virulence in vivo

**Fig 6:**
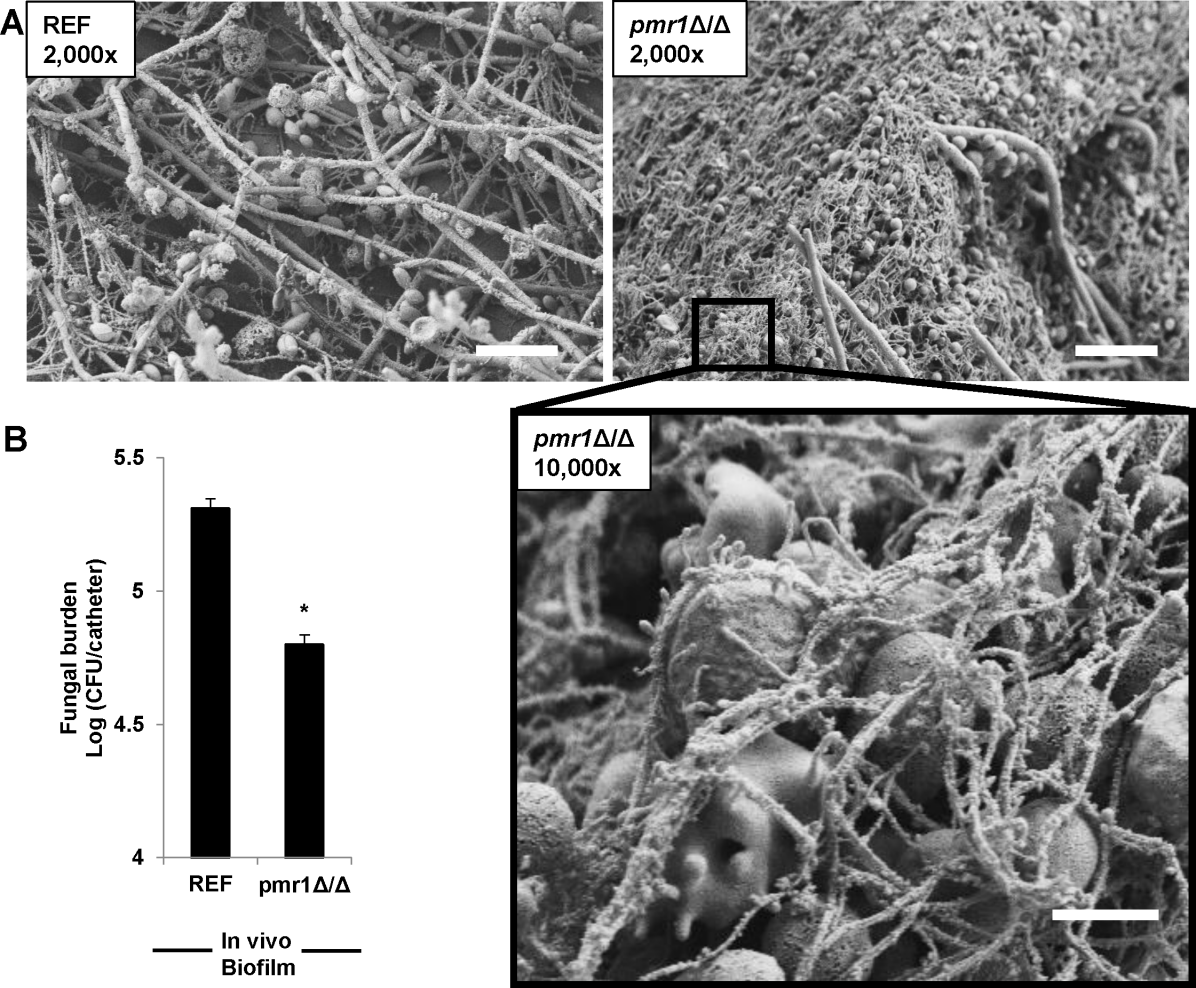
*C*. *albicans* biofilm mannosylation impairs NET release and contributes to virulence in vivo. (A) *C*. *albicans* biofilms were formed in rat venous catheters in vivo and imaged by scanning electron microscopy. While both the reference strain and the *pmr1*Δ/Δ mutant formed biofilms in vivo, the *pmr1*Δ/Δ mutant biofilm was associated with increased host cells and overlying fibrillary material. On higher magnification, the web of fibrils appeared to extrude from host cells. (B) The contribution of mannosylation by *PMR1* to biofilm immune protection was assessed by rat catheter viable burden. Disruption of *PMR1* was associated with a 70% decrease in burden compared to the reference strain. *P<0.*05*.

To assess the role of mannosylation and matrix production on neutrophil function in vivo, we utilized a rat vascular catheter model of biofilm infection where biofilms are propagated on jugular venous catheters following luminal inoculation [37, 38]. We analyzed biofilm architecture and host response with scanning electron microscopy. The reference strain produced a confluent layer of yeast and hyphae, as has been previously described (Fig 6A) [37, 38]. The *pmr1*Δ/Δ mutant also produced a robust biofilm in vivo. However, many host cells were found associating with the biofilm and it was covered with numerous thread-like structures. On higher magnification, these fibrils were seen protruding from a host cell, ultimately forming a web, consistent with the release of NETs. This fibrillary material was less apparent for the reference strain biofilm.

We next asked if the increase NET release in response to the *pmr1*Δ/Δ mutant correlated with increase fungal killing. Infected rat venous catheters were collected for viable burden determination. Compared to the reference strain, the *pmr1*Δ/Δ mutant exhibited a 70% reduction in microbial burden (Fig 6B). This corroborates in vitro studies demonstrating increased killing of the *pmr1*Δ/Δ mutant biofilm. Strikingly, the phenotype was even more pronounced in vivo. The studies show that mannosylation of *Candida* biofilms inhibits NETs, leading to decreased killing in vivo.

## Discussion

The propensity of *Candida* to form biofilms provides a means for it to withstand host defenses and antifungal therapies, a major obstacle for effective treatment [4, 39, 40]. Neutrophils are essential for immunity to many invasive fungal infections including candidiasis [15–18]. In response to many pathogens, including those too large to by phagocytosed, these cells release NETs which entrap and kill various microorganisms, including *C*. *albicans* [21–23]. Prior reports have revealed that *C*. *albicans* biofilms resists killing by neutrophils, an essential leukocyte for immunity to invasive fungal infections [6, 7, 25]. Here we show that unlike planktonic *C*. *albicans*, biofilms fail to trigger the release of NETs. This manner of avoiding neutrophil killing is unique to the biofilm lifestyle and sheds light on why *Candida* biofilms are so resilient.

This study provides multiple, complementary lines of evidence that the impairment of NET release in response to *C*. *albicans* biofilms is linked to the presence of an extracellular matrix. First, dispersion of microtiter plate biofilms interrupted the inhibitory process and permitted NET release. Likewise, disruption of biofilms growing on a coverslips triggered NETs. Additionally, multiple *C*. *albicans* mutants lacking a mature extracellular matrix elicited NETs. The increase in NETs correlated with susceptibility to neutrophil killing both in vitro and in vivo in a rat venous catheter model of biofilm infection. Furthermore, we showed that neutrophils pre-stimulated to induce NETs are active against biofilms, highlighting the importance of this inhibitory pathway. Together, these studies show the *C*. *albicans* biofilm matrix inhibits NET release, which contributes to immune evasion and provides a survival advantage.

Recent investigations have provided a refined characterization of the *C*. *albicans* extracellular matrix, identifying polysaccharide structures distinct from those found in the cell wall [11, 14]. The most abundant matrix polysaccharide, α-mannan, was in structures over 50-fold larger than those described for mannan of the cell wall [12]. A screen for genetic regulators of matrix mannan production identified 7 genes involved in mannan pathways [14]. These genes served roles in production or delivery of matrix mannan, biofilm architecture, and biofilm-associated drug tolerance via antifungal sequestration. Our current investigation identified 4 of these same mannan genes in a screen designed to find genes regulating the biofilm inhibition of NETs (*ALG11*, *MNN9*, *PMR1*, and *VRG4*). These findings suggest that the core group of genes responsible for matrix production and drug tolerance also contribute to resistance to neutrophil attack.

One limitation of this study is that genetic disruption of *Candida* matrix production can also impact the cell wall integrity, as the machinery for these two processes is overlapping [14]. For example, during biofilm growth, *PMR1* is required for production of extracellular matrix polysaccharides, including a large mannan structure which ultimately associates with glucan [14]. However, disruption of *PMR1* also impacts both O- and N-mannan, resulting in truncated cell wall mannans [31]. Our current studies identify a phenotype for the *pmr1Δ/Δ* mutant during biofilm formation, consistent with disruption of matrix, a biofilm-specific process. Differences in the triggering of NETs or the susceptibility to neutrophils were not observed between the *pmr1Δ/Δ* mutant and reference strain during planktonic growth. In addition, disruption of matrix by dispersion of the intact biofilm also mimicked the phenotype.

In our studies, the *C*. *albicans* biofilm inhibition of NETs correlated mechanistically with dampened neutrophil ROS. Biofilm alone did not induce neutrophil ROS above the baseline and completely inhibited PMA induction of ROS. Conversely, the *pmr1Δ/Δ* biofilm, which triggered NETs, also elicited neutrophil ROS. A similar pattern of ROS release was observed in response to dispersed biofilm. Both of these observations are consistent with a role for biofilm matrix for this process. Most NET pathways involve NADPH-oxidase produced ROS with processing by myeloperoxidase, including NET induction by *C*. *albicans* [33, 41, 42]. In the current study, *C*. *albicans* biofilm suppression of both ROS and NET release was not overcome by PMA activation of protein kinase C, suggesting the presence of an inhibitory pathway of NADPH-oxidase which is downstream of protein kinase C. Interestingly, a process of ROS-independent rapid NET release has been observed for *Candida* when primed neutrophils are presented with fibronectin [43, 44]. We considered the possibility of this pathway in response to *C*. *albicans* biofilm, but NET release was not identified by Sytox green staining or scanning electron microscopy imaging at early time points (15 min-1 h).

Multiple moieties on the cell wall of planktonic *Candida* have been shown to be involved in phagocyte recognition, including *N-* and *O*-linked mannans, β-glucans, and chitin [18]. Investigations have begun to explore a role for receptors recognizing these pathogen-associated molecular patterns (PAMPs) in NETosis [22, 43, 45]. The induction of NETs in response to immobilized β-glucan and fibronectin was found to be dependent on β-glucan recognition by complement receptor 3, but not by Dectin-1 [43]. In contrast, studies examining the NET response to the fungal pathogen *Paracoccidioides brasiliensis* discovered a key role for dectin-1 [45]. In *C*. *albicans*, this receptor has been shown to modulate NETosis [22]. Upon phagocytosis of a mutant *C*. *albicans*, which was “yeast-locked” and unable to filament, dectin-1 was implicated in negative regulation of NETosis [22]. In the current investigation, we show impaired NET release upon exposure to *C*. *albicans* biofilm. One intriguing possibility is that the biofilm matrix is masking cell wall epitopes important for neutrophil recognition and NET production. Alternatively, a unique epitope in the matrix material may prompt activation of a pathway distinct from NETosis. It is also interesting to postulate the NET response to alternative *Candida* biofilms. For example, biofilms with a less dense, more permeable matrix, such sexual biofilms with mating type locus a/a or α/α, may be expected allow for higher NETosis [46, 47].

Prior investigations have identified diverse mechanisms of resisting NETs for a variety of pathogens. One of the intriguing mechanisms of NET evasion for bacterial pathogens is the production of extracellular deoxyribonucleases with the capacity to degrade NETs [48–53]. Enzymes produced by bacteria, such a catalase, are also protective through degradation of NET-associated reactive oxygen species [54]. Several mechanisms account for NET evasion for *Aspergillus* [55]. *A*. *fumigatus* conidia express a hydrophobin (RodA) which suppresses NET formation [55, 56]. The hyphal forms appear to be protected from NETs by galactosaminogalactan, a positively charged exopolysaccharide which is proposed to inhibit binding of NET components [56]. For *Cryptococcus spp*., the surrounding capsule modulates the neutrophil response [57]. For example, *C*. *gattii* capsular components can induce NET release, but are protective from NET killing [57, 58]. In contrast, the capsular polysaccharide glucuronoxylomannan of *C*. *neoformans* inhibits the production of NETs [58]. Mechanisms of NET inhibition for *Candida spp*. have not previously been described. To our knowledge, the present study represents the first study showing the inhibition of NET release in a biofilm-specific manner.

We and others have shown that *Candida* biofilms are many fold more resistant to neutrophil killing when compared to planktonic cells [7, 25, 26]. Here we describe a pronounced impairment of NET release in response to biofilm which accounts for resistance to neutrophils. However, as disruption of the biofilm matrix did not completely reverse this phenotype or elicit NET formation to the level of planktonic cells, other mechanisms are likely in play as well. In addition, the inhibition of NETs may have more broad implications beyond providing protection from neutrophil killing. In vivo, these NETs may play a critical role in preventing dissemination to distant sites, unmasking epitopes for fungal recognition, or recruiting additional inflammatory cells [59, 60]. A role for NETs in leukocyte recruitment would provide an answer to why the difference in killing between the *pmr1Δ/Δ* biofilm the reference strain was greater in vivo.

Our studies identify a mechanism of neutrophil impairment by *C*. *albicans* that is unique to biofilm formation. Our data show how biofilm extracellular matrix alters neutrophil recognition of *Candida*, inhibiting release of NETs and neutrophil associated fungal killing. Further understanding of this pathway may assist in the development of strategies to augment the host immune response to biofilm infections.

## Materials and Methods

### Organisms and inoculum

*C*. *albicans* strains used in this study are listed in S1 Table. The library of mutants with disruption of mannan and glucan genes has previously been described [14]. *C*. *albicans* SC5314 was used as the wild type strain and SN250 was used as the reference strain for mutants [14, 61, 62]. Strains were stored in 15% (vol/vol) glycerol stock at -80°C and maintained on yeast extract-peptone-dextrose (YPD) medium + uridine (1% yeast extract, 2% peptone, 2% dextrose, and 80 μg/ml uridine) prior to experiments. Cultures were propagated overnight in YPD supplemented with uridine at 30°C on an orbital shaker at 200 RPM. For biofilm experiments, *C*. *albicans* was resuspended in RPMI-MOPS at a concentration of 1.5 x 10^6^ cell/ml and 200 μl was added to wells of 96-well plates followed by a 24 h incubation at 37°C, unless otherwise specified [63]. For experiments using planktonic *Candida*, 1 ml of an overnight culture was inoculated into 20 ml of fresh YPD broth and incubated at 30°C on an orbital shaker at 200 RPM for 2 hours, washed twice with phosphate buffered saline (-calcium, -magnesium) DPBS (Hyclone Laboratories Inc., Logan, UT), and enumerated by hemocytometer. To assess biofilm burden and determine an equivalent burden of planktonic organisms, an XTT (2,3-Bis-(2-Methoxy-4-Nitro-5-Sulfophenyl)-2H-Tetrazolium-5-Carboxanilide) assay was performed as an estimate of viable burden [63]. Background absorbance at 492 nm was recorded prior to the addition of XTT and subtracted from final readings. Results showed a burden of 1.5 x 10^6^ planktonic cells/well to be similar to the biofilm burden (S2 Fig). Therefore, this number of planktonic cells was used in neutrophil co-culture experiments comparing the response to biofilm and planktonic cells [63].

### Human neutrophil collection

Blood was obtained from volunteer donors with written informed consent through a protocol approved by the University of Wisconsin Internal Review Board (IRB). Primary human neutrophils were purified by negative antibody selection using the MACSxpress Neutrophil Isolation and MACSxpress Erythrocyte Depletion kits (Miltenyi Biotec Inc., Auburn, CA). Experiments with neutrophils were performed in RPMI 1640 (without phenol red) supplemented with 2% heat-inactivated fetal bovine serum (FBS) and supplemented with glutamine (0.3 mg/ml). Incubations were at 37°C with 5% CO_2_.

### Sytox Green assays

As a measure of NET release, a Sytox Green assay was adapted for use in biofilm assays [27]. *C*. *albicans* biofilms were grown in wells of 96-well opaque plates and neutrophils were added to a final concentration of 2x10^5^ cell/well. After a 4 h incubation, Sytox Green (Life Technologies, Eugene, OR) was added at a final concentration of 1 μM and fluorescence (excitation 500 nm/emission 528 nm) was measured in an automated plate reader. To reduce the background signal, plates were read immediately following the addition of Sytox Green. Background signals on average were 9% of positive controls. For a subset of experiments, PMA (100 nM) was included. For experiments involving biofilms supernatants, *C*. *albicans* biofilms were grown in 6-well plates for 24 hours and washed twice with DPBS prior to addition of fresh media for 2 h. Supernatants were collected and centrifuged at 1200x*g* at 4°C for 20 min. For experiments with dispersed biofilms, biofilms were physically dispersed by gentle pipetting. A similar process for planktonic cells did not impact the NET response by Sytox Green (S3 Fig). Experiments using planktonic cells were similarly performed by adding and allowing 1.5 x 10^6^ cells/well to settle prior to the addition of neutrophils. Background fluorescence for each condition was subtracted from total fluorescence values.

### Measurement of ROS

For measurement of neutrophil ROS production, an oxidative stress assay was adapted for use in biofilm [64, 65]. Briefly, neutrophils were stained with the fluorescent dye CMH(2)CFDA (Life Technologies, Eugene, OR) in DPBS for 10 min at room temperature in the dark and added to biofilms growing in 96-well opaque plates to final concentration of 2x10^5^ neutrophils/well. Fluorescence (excitation 495 nm; emission 527 nm) was recorded every 30 min for 3 h and data are shown for 2.5 hours, as this represented the max reading, prior to over reads or decline. Experiments using planktonic cells were similarly performed with the addition of 1.5 x 10^6^ cells/well. Background fluorescence was determined for each *C*. *albicans* condition and subtracted from total fluorescence values prior to data analysis.

### Killing assays

Briefly, an XTT (2,3-bis-(2-methoxy-4-nitro-5-sulfophenyl)-2H-tetrazolium-5-carboxanilide) metabolic assay was used to estimate *C*. *albicans* viability following co-culture with neutrophils [6, 63]. Following a 12 or 24 h incubation period, biofilms were washed with DPBS. To compare the killing of biofilm and planktonic cells, 24 h biofilms were compared to planktonic cells at a concentration of 1.5 x 10^6^ cells/well. Neutrophils were added to a final concentration of 1.5 x 10^6^ cells/well (effector:target of 1:1). For a subset of experiments, PMA 100 nM was included in select wells to induce NETs [21]. Following a 5 h incubation, neutrophils were lysed for 20 minutes at 37°C in TritonX-100 at a final concentration of 0.03% with 50 RPM agitation. Following lysis, 90 μL of 9:1 XTT working solution (0.75 mg/ml XTT in PBS with 2% glucose: phenazine methosulfate 0.32 mg/ml in ddH2O) was added to each well. After a 25 min incubation, samples were transferred to a Falcon 96 well U bottom plate and centrifuged at 1,200×g to pellet cells. Supernatants (110 μl) were then transferred to a 96 well flat bottom plate for absorption reading at 492 nm. To determine percent killing, values were compared to wells without neutrophils after subtraction of baseline absorbance. A subset of experiments was similarly performed using 12 h biofilms and 1.5 x 10^6^ neutrophils/well to examine the impact of pre-stimulation of NETs and disruption of *PMR1*.

### Microfluidic device model and imaging

For microfluidic experiments, biofilms were grown in straight channels of a microfluidic device (Iuvo Microchannel 5250, Thermo Fisher). Channels were pretreated with fibrinogen 10 μg/ml in DPBS for 1 h prior and rinsed three times with RPMI-MOPS prior to loading 2 μl *C*. *albicans* at a concentration of 10^6^ cells/ml. The plate was incubated for 1 h at room temperature on its vertical access to allow for settling and adherence to the sidewall. The plate was incubated for an additional 18 h vertically at 37°C. Biofilms were then gently washed 3 times with RPMI supplemented with 2% FBS and 3 μl of fluorescently labeled human neutrophils at 2×10^6^ cells/ml were added. These cells had previously been stained with Calcein AM (ThermoFischer Scientific, Waltham, MA) at 0.5 μg/ml in DPBS for 10 min at room temperature in the dark. *Candida*-neutrophil interactions were analyzed by time lapse microscopy with microfluidic devices incubating at 37°C. For imaging of the initial (0–90 min) interaction with biofilms, images were obtained every 60 sec using bright field and fluorescence (excitation 480 nm, emission 525nm) at 10x for 90 minutes on an inverted microscope (Nikon Eclipse TE300) equipped with a motorized stage (Ludl Electronic Products), charge-coupled device camera (CoolSNAP ES2), and MetaVue imaging software v6.2. Images and videos were compiled using ImageJ. Video is shown at 5 frames per second. For imaging of neutrophil viability (3–5 h), Calcein AM-labeled neutrophils were incubated with *Candida* biofilms for 3 h prior to imaging. After the addition of 4 μl propidium idodide (3 μM), devices were incubated for an additional 15 min. Images were then obtained every 2 min using bright field and fluorescence (excitation 480 nm, emission 525nm and excitation 565, emission 620) for 2 h and compiled at 7 frames per second. Experiments were similarly performed with planktonic *C*. *albicans* in glass coverslip bottom petri dishes (MatTek, Ashland, Ma).

### Immunofluorescent imaging

As a qualitative measure of NETs, immunofluorescent imaging for histone citrullination was performed. Biofilms were grown in glass coverslip bottom petri dishes (MatTek, Ashland, Ma) or two channel microfluidic devices (Beebe lab, UW-Madison) pre-treated with 10μg/ml fibrinogen (Sigma-Aldrich, St. Louis, MO) in 0.1% gelatin (Millipore, Temecula, CA). Biofilms were washed 3 times after 22 h of growth and neutrophils were added at a concentration of 2×10^6^ cells/ml. After 4 h, biofilms were fixed with 4% formaldehyde in DPBS for an additional 4 h. Fixed co-cultures were rinsed 3×5 min with DPBS and incubated with antibody blocking buffer (2% w/v bovine serum albumin (BSA) and 0.02% v/v Tween 20 in PBS) overnight at 4°C. All steps were performed very gently to preserve NETs. Following rinsing with antibody binding buffer (0.1% BSA w/v and 0.005% v/v Tween 20 in PBS), primary antibody (anti-histone H4, citrulline3) in antibody binding buffer at 1:1000 was added for 2 h at room temperature [29]. Samples were rinsed gently 6× 5 min and secondary antibody (goat-anti rabbit IgG Fc DyLight 594 conjugated) at 1:200 in antibody binding buffer was added for a 2 h incubation in the dark. Samples were rinsed 6×5 min with antibody binding buffer and brightfield and fluorescent (excitation 565, emission 620) images were obtained using the 20x objective on an inverted microscope (Nikon Eclipse TE300) equipped with a charge-coupled device camera (CoolSNAP ES2) and MetaVue imaging software v6.2. Images were processed using ImageJ.

### Biofilm coverslip model

A coverslip model of biofilm formation was adapted for use with neutrophils [14]. Briefly, *C*. *albicans* resuspended in RPMI-MOPS at 10^6^ cells/ml was added to poly-L-lysine coated coverslips (13 mm, Thermonax plastic for cell culture) for 30 min at 30°C. After the adherence period, non-adherent cells were removed and fresh RPMI-MOPS was added. Biofilms were grown for 24 h at 37°C on an orbital shaker at 50 RPM and washed with DPBS. Neutrophils (5 x 10^5^) were added and coverslips were collected for scanning electron microscopy, as described below, after 15 min, 1 h, or 4 h.

### In vivo venous catheter biofilm model

Specific-pathogen-free Sprague-Dawley rats weighing 350 g (Harlan Sprague-Dawley, Indianapolis, Ind.) were used for all studies. A jugular vein rat central venous catheter biofilm infection model was used as previously described [38, 66]. Briefly, 24 h following surgical jugular venous catheter insertion, *C*. *albicans* at 10^6^ cells/ml was instilled in the catheter lumens. After 24 h, the inocula were removed and catheters were harvested and collected for imaging by scanning electron microscopy, as described below, or viable burden measurement. Viable burdens were determined by plating of serial dilutions on Sabouraud Dextrose Agar in triplicate.

### Scanning electron microscopy

Rat vascular catheter biofilms were processed and imaged by scanning electron microscopy as previously described [38]. Coverslips of biofilm and planktonic co-culture with neutrophils were similarly processed. Briefly, after washing with DPBS, samples were fixed overnight (4% formaldehyde, 1% glutaraldehyde, in PBS). They were then washed with PBS, treated with 1% osmium tetroxide, and washed again. Samples were dehydrated through series of ethanol washes followed by critical point drying and mounted on aluminum stubs. Following sputter coating with either gold or platinum, samples were imaged in a scanning electron microscope (LEO 1530) at 3kV.

### RNA collection and quantitative RT-PCR

RNA was purified from 24 h biofilms and planktonic cells using the RNeasy Minikit (Qiagen) and quantified using a NanoDrop spectrophotometer [67]. TaqMan primer and probe sets were designed as previously described [67]. Primers for *ACT1* included AGCTTTGTTCAGACCAGCTGATT (ACT1 RT For), GGAGTTGAAAGTGGTTTGGTCAA (ACT1 RT Rev), and /56-FAM/CCAGCAGCTTCCAAACCT/36-TAMSp/ (ACT 1 Probe). Primers for *PMR1* included TGGATGGGCAATAAGGATGAC (PMR1 RT For), TGTTAACTTAGCCTCTGCAGG (PMR1 RT Rev), and /56-FAM/AGTGATGGCTAATGTGATCGATATGGCG/36-TAMSp/ (PMR1 Probe). The QuantiTect probe RT-PCR kit (Qiagen) was used in a CFX96 real-time PCR detection system (Bio-Rad) with the following program: 50°C for 20 min, initial denaturation at 95°C for 15 min, and then 40 cycles of 94°C for 15 s and 55°C for 1 min. Reactions were performed in triplicate. The quantitative data analysis was completed using the delta-delta *CT* method [68]. The comparative expression method generated data as transcript fold-change normalized to a constitutive reference gene transcript (*ACT1*) and as transcript fold-change relative to planktonic cells.

### Statistics

Experiments were performed at least 3 times using neutrophils from different donors on different days. Statistical analyses were performed by Student’s t-test using Sigma Stat or Excel software. Differences of P<0.05 were considered significant.

### Ethics statement

For studies with neutrophils from human subjects, written informed consent was obtained from healthy donors at the time of blood draw with the approval of the University of Wisconsin-Madison Center for Health Sciences Human Subjects Committee. All animal procedures were approved by the Institutional Animal Care and Use Committee at the University of Wisconsin according to the guidelines of the Animal Welfare Act, The Institute of Laboratory Animal Resources Guide for the Care and Use of Laboratory Animals, and Public Health Service Policy under protocol MV1947.

## Supporting Information

S1 Fig Biofilm formation by *C*. *albicans* mannan mutants. (A) Biofilm formation after 24 h was measured by XTT assay. The *pmr1Δ/Δ* mutant formed a biofilm with burden similar to the reference strain, *n = 3*, *SEM shown*. *P<0.*05*. (B) *C*. *albicans* biofilms were growth for 24 h, processed, and imaged by scanning electron microscopy at 2000x. Biofilms formed by *pmr1Δ/Δ* and the reference stain had a similar appearance, consisting of a dense mat of hyphae.
(PDF)

S2 Fig Biofilm and planktonic normalization. The burden of 24 h biofilms was measured by XTT assay and compared to various concentrations of planktonic cells to determine a similar burden for studies, *n = 3*, *SEM shown*
(PDF)

S3 Fig Impact of dispersion process on planktonic cells. Planktonic *C*. *albicans* cells were dispersed by gentle pipetting, mimicking the disruption process for biofilms, and then co-cultured with human neutrophils for 4 h and NET release was estimated by Sytox Green detection of free DNA, *n = 3*, *SEM shown*.
(PDF)

S1 Table Strains used in the study. (DOCX)

S2 Table Screen for NET induction in response to *C*. *albicans* mutant biofilms. (DOCX)

S1 File Neutrophils adhere to *C*. *albicans* biofilm. Fluorescently labeled neutrophils were added to microfluidic channels with *C*. *albicans* biofilms, which had been propagated on the sidewall. Bright field and fluorescent images were collected every 60 s and compiled at 5 frames per second. Over the course of 90 min, neutrophils migrated to the biofilm, adhered, and extended over the surface of the hyphae.
(MP4)

S2 File Neutrophils engulf planktonic *C*. *albicans*. Fluorescently labeled neutrophils were added to planktonic *C*. *albicans* cells. Bright field and fluorescent images were collected every 60 s and compiled at 5 frames per second. Over the course of 90 min, neutrophils phagocytosed the planktonic cells.
(MP4)

S3 File Neutrophils adherent to *C*. *albicans* biofilm remain viable. Calcein AM-labeled neutrophils (green) were added to microfluidic channels with *C*. *albicans* biofilms, which had been propagated on the sidewall. After a 3 h incubation, propidium iodide was added. Bright field and fluorescent images were then collected every 2 min for 2 h and compiled at 7 frames per second. The vast majority of neutrophils remained viable during this time period, actively migrating and adhering to hyphae. Very few cells were dead, as marked by propidium iodide staining (red). In addition, the biofilm cells excluded the dye, consistent viability. Extracellular propidium iodide staining to suggest the presence of NETs was not observed.
(MP4)
