# Supplementary figures and images for: The Extracellular Matrix of Candida albicans Biofilms Impairs Formation of Neutrophil Extracellular Traps
Source: PLoS Pathog. 2016 Sep 13;12(9):e1005884. doi: 10.1371/journal.ppat.1005884 (PMC5021349; doi:10.1371/journal.ppat.1005884)

**A**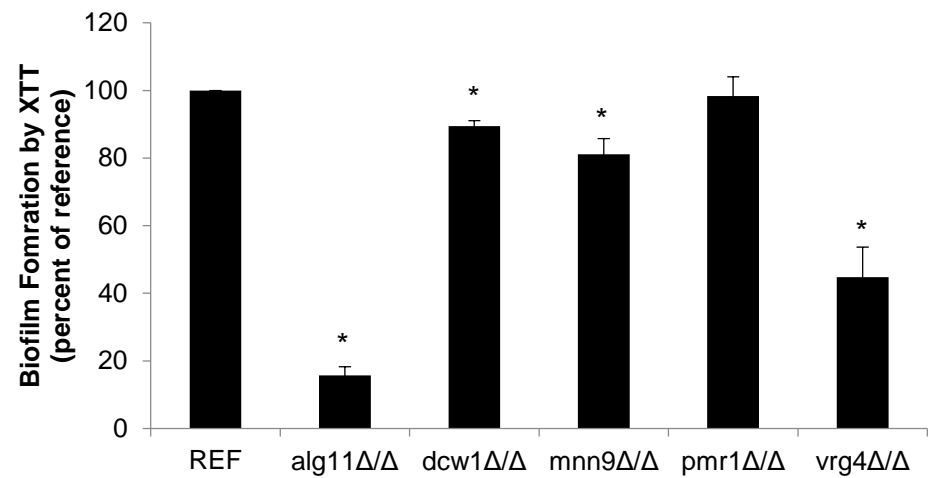**B**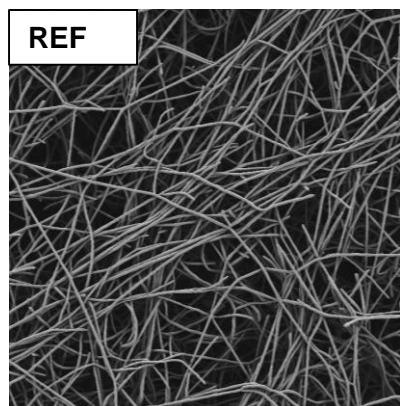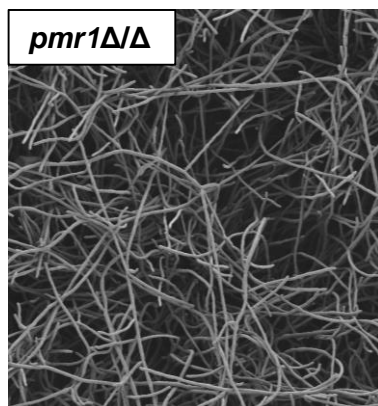

Supplement: S1 Fig — (A) Biofilm formation after 24 h was measured by XTT assay. The pmr1Δ/Δ mutant formed a biofilm with burden similar to the reference strain, n = 3, SEM shown. *P<0.05. (B) C. albicans biofilms were growth for 24 h, processed, and imaged by scanning electron microscopy at 2000x. Biofilms formed by pmr1Δ/Δ and the reference stain had a similar appearance, consisting of a dense mat of hyphae. (PDF) [file ppat.1005884.s001.pdf]

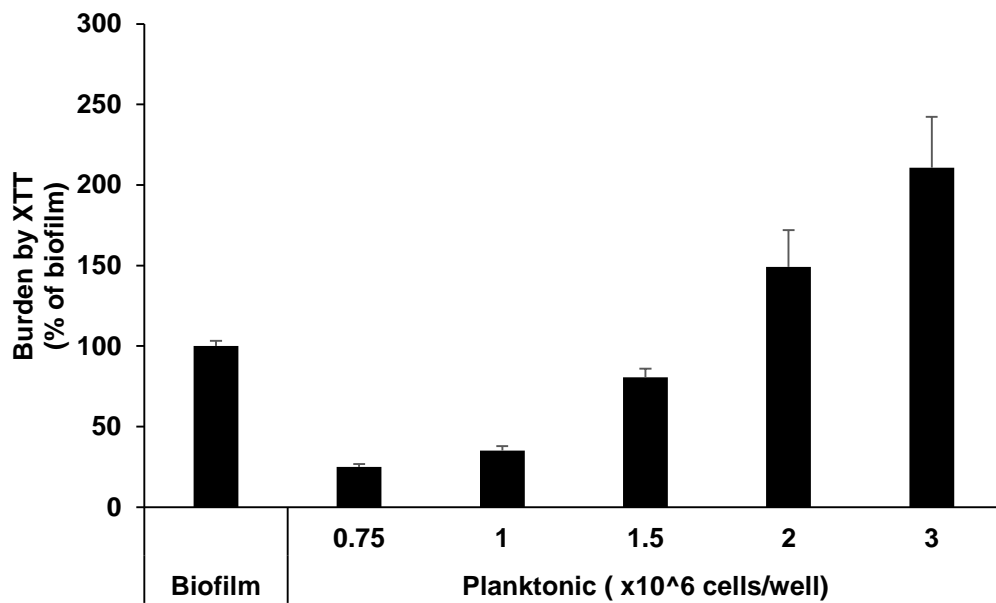

Supplement: S2 Fig — The burden of 24 h biofilms was measured by XTT assay and compared to various concentrations of planktonic cells to determine a similar burden for studies, n = 3, SEM shown (PDF) [file ppat.1005884.s002.pdf]

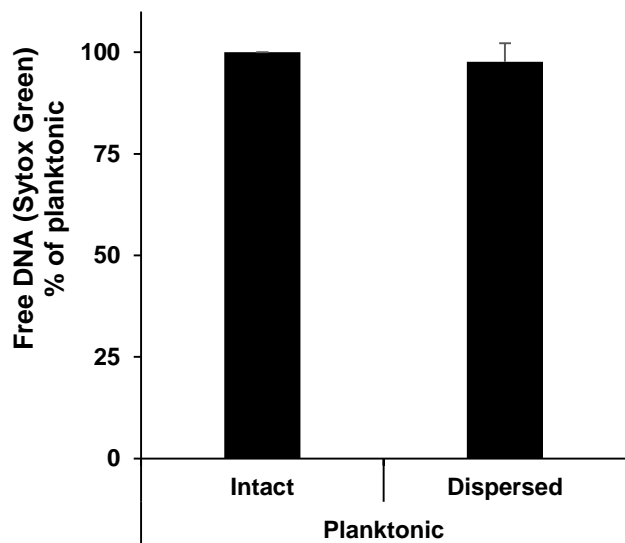

Supplement: S3 Fig — Planktonic C. albicans cells were dispersed by gentle pipetting, mimicking the disruption process for biofilms, and then co-cultured with human neutrophils for 4 h and NET release was estimated by Sytox Green detection of free DNA, n = 3, SEM shown. (PDF) [file ppat.1005884.s003.pdf]
