# Supplementary material for: The Extracellular Matrix of Candida albicans Biofilms Impairs Formation of Neutrophil Extracellular Traps
Source: PLoS Pathog. 2016 Sep 13;12(9):e1005884. doi: 10.1371/journal.ppat.1005884 (PMC5021349; doi:10.1371/journal.ppat.1005884)
Supplement: S1 Table — (DOCX) [file ppat.1005884.s004.docx]

**S1 Table. Strains used in this study**

| **Gene Name** | **Genotype** | **Strain Name** | **Description*** | Ref |
| --- | --- | --- | --- | --- |
|  |  | SS5314 | Wildtype | [1] |
|  |  | SN250 | Reference strain | [2] |
| *MNN2* | Δ/Δ | KMR391 | α-1,2 mannosyltransferase | [3] |
| *MNN21* | Δ/Δ | KFM148 | α-1,2 mannosyltransferase | [3] |
| *MNN22* | Δ/Δ | KFM132 | α-1,2 mannosyltransferase | [3] |
| *MNN23* | Δ/Δ | KMR350 | α-1,2 mannosyltransferase | [3] |
| *MNN24* | Δ/Δ | KFM113 | α-1,2 mannosyltransferase | [3] |
| *MNN26* | Δ/Δ | KMR383 | α-1,2 mannosyltransferase | [3] |
| *ALG11* | Δ/Δ | URZ217 | α-1,2 mannosyltransferase | [3] |
| *MNN9* | Δ/Δ | KMR392 | α-1,6 mannosyltransferase | [3] |
| *MNN10* | Δ/Δ | KMR338 | α-1,6 mannosyltransferase | [3] |
| *MNN11* | Δ/Δ | KMR347 | α-1,6 mannosyltransferase | [3] |
| *HOC1* | Δ/Δ | ELR117 | α-1,6 mannosyltransferase | [3] |
| *OCH1* | Δ/Δ | ELR125 | α-1,6 mannosyltransferase | [3] |
| *VAN1* | Δ/Δ | ELR108 | α-1,6 mannosyltransferase | [3] |
| *ANP1* | Δ/Δ | ELR107 | α-1,6 mannosyltransferase | [3] |
| *MNT4* | Δ/Δ | KMR336 | mannosylphosphate transferase | [3] |
| *MNN4* | Δ/Δ | KFM138 | mannosylphosphate transferase | [3] |
| *MNN4-4* | Δ/Δ | KMR384 | mannosylphosphate transferase | [3] |
| *MNN41* | Δ/Δ | KMR351 | mannosylphosphate transferase | [3] |
| *MNN42* | Δ/Δ | KMR349 | mannosylphosphate transferase | [3] |
| *MNN44* | Δ/Δ | KMR380 | mannosylphosphate transferase | [3] |
| *MNN46* | Δ/Δ | KMR386 | mannosylphosphate transferase | [3] |
| *MNN47* | Δ/Δ | KMR341 | mannosylphosphate transferase | [3] |
| *MNT3* | Δ/Δ | KMR364 | mannosylphosphate transferase | [3] |
| *MNT5* | Δ/Δ | ELR118 | mannosylphosphate transferase | [3] |
| *DCW1* | Δ/Δ | ELR127 | mannosidase | [3] |
| *DFG5* | Δ/Δ | ELR129 | mannosidase | [3] |
| *AMS1* | Δ/Δ | ELR115 | mannosidase | [3] |
| *MNS1* | Δ/Δ | KFM107 | mannosidase | [3] |
| *VRG4* | Δ/Δ | URZ192 | GDP-mannose transporter | [3] |
| *PMR1* | Δ/Δ | KMR355 | Ca^2+^/Mn^2+^ ATPase | [3] |
| *PMR1* | Δ/Δ, + | KFM150 | Ca^2+^/Mn^2+^ ATPase | [3] |
| *KRE1* | Δ/Δ | URZ266 | β-1,6 glucan synthesis | [3] |
| *KRE5* | Δ/Δ | JAE104 | β-1,6 glucosyltransferase | [3] |
| *KRE6* | Δ/Δ | URZ270 | β-1,6 glucan synthesis | [3] |
| *KRE9* | Δ/Δ | JAE101 | β-1,6 glucan synthesis | [3] |
| *BIG1* | Δ/Δ | JAE103 | β-1,6 glucan synthesis | [3] |
| *SKN1* | Δ/Δ | URZ260 | β-1,6 glucan synthesis | [3] |

1. Gillum AM, Tsay EY, Kirsch DR. Isolation of the *Candida albicans* gene for orotidine-5'-phosphate decarboxylase by complementation of *S. cerevisiae* ura3 and *E. coli* pyrF mutations. Mol Gen Genet. 1984; 198**:** 179-182.

2. Noble SM, Johnson AD. Strains and strategies for large-scale gene deletion studies of the diploid human fungal pathogen *Candida albicans*. Eukaryot Cell. 2005; 4**:** 298-309.

3. Mitchell KF, Zarnowski R, Sanchez H, Edward JA, Reinicke EL, Nett JE, et al. Community participation in biofilm matrix assembly and function. Proc Natl Acad Sci U S A. 2015; 112**:** 4092-4097.
