# Supplementary material for: The Extracellular Matrix of Candida albicans Biofilms Impairs Formation of Neutrophil Extracellular Traps
Source: PLoS Pathog. 2016 Sep 13;12(9):e1005884. doi: 10.1371/journal.ppat.1005884 (PMC5021349; doi:10.1371/journal.ppat.1005884)
Supplement: S2 Table — (DOCX) [file ppat.1005884.s005.docx]

**S2 Table. Screen for NET induction in response to *C. albicans* mutant biofilms.**

| **Gene Name** | **Fold change from reference**  **(Sytox Green)** | **Description** |
| --- | --- | --- |
|  | 1 | Reference strain-SN250 |
| **Mannan** |  |  |
| *alg11*Δ/Δ | 9.2 | α-1,2 mannosyltransferase |
| *vrg4* Δ/Δ | 5.95 | GDP-mannose transporter |
| *pmr1*Δ/Δ | 4.29 | Ca^2+^/Mn^2+^ ATPase |
| *dcw1*Δ/Δ | 2.29 | mannosidase |
| *ams1*Δ/Δ | 2.2 | mannosidase |
| *mnn9*Δ/Δ | 1.68 | α-1,6 mannosyltransferase |
| *och1*Δ/Δ | 1.66 | α-1,6 mannosyltransferase |
| *mnn11*Δ/Δ | 1.65 | α-1,6 mannosyltransferase |
| *mnn47*Δ/Δ | 1.64 | mannosylphosphate transferase |
| *mnn41*Δ/Δ | 1.52 | mannosylphosphate transferase |
| *van1*Δ/Δ | 1.33 | α-1,6 mannosyltransferase |
| *mnn4*Δ/Δ | 1.17 | mannosylphosphate transferase |
| *anp1*Δ/Δ | 1.15 | α-1,6 mannosyltransferase |
| *mnt4*Δ/Δ | 1.13 | mannosylphosphate transferase |
| *hoc1*Δ/Δ | 1.12 | α-1,6 mannosyltransferase |
| *mnn44*Δ/Δ | 1.09 | mannosylphosphate transferase |
| *mnn4-4*Δ/Δ | 1.06 | mannosylphosphate transferase |
| *mnn23*Δ/Δ | 1.04 | α-1,2 mannosyltransferase |
| *mnt5*Δ/Δ | 1.02 | mannosylphosphate transferase |
| *mnn2*Δ/Δ | 1 | α-1,2 mannosyltransferase |
| *mnn42*Δ/Δ | 0.99 | mannosylphosphate transferase |
| *mnn24*Δ/Δ | 0.98 | α-1,2 mannosyltransferase |
| *mnn21*Δ/Δ | 0.96 | α-1,2 mannosyltransferase |
| *mnn46*Δ/Δ | 0.89 | mannosylphosphate transferase |
| *mnt3*Δ/Δ | 0.85 | mannosylphosphate transferase |
| *mnn22*Δ/Δ | 0.85 | α-1,2 mannosyltransferase |
| *mns1*Δ/Δ | 0.81 | mannosidase |
| *mnn26*Δ/Δ | 0.81 | α-1,2 mannosyltransferase |
| *dfg5*Δ/Δ | 0.65 | mannosidase |
| *mnn10*Δ/Δ | 0.57 | α-1,6 mannosyltransferase |
| **β-1,6 glucan** |  |  |
| *big1*Δ/Δ | 4.23 | β-1,6 glucan synthesis |
| *kre5*Δ/Δ | 3.42 | β-1,6 glucosyltransferase |
| *skn1*Δ/Δ | 2.36 | β-1,6 glucan synthesis |
| *kre9*Δ/Δ | 2 | β-1,6 glucan synthesis |
| *kre1*Δ/Δ | 1.24 | β-1,6 glucan synthesis |
| *kre6*Δ/Δ | 0.78 | β-1,6 glucan synthesis |
